# Crowdsourcing for Cognitive Science – The Utility of Smartphones

**DOI:** 10.1371/journal.pone.0100662

**Published:** 2014-07-15

**Authors:** Harriet R. Brown, Peter Zeidman, Peter Smittenaar, Rick A. Adams, Fiona McNab, Robb B. Rutledge, Raymond J. Dolan

**Affiliations:** 1 Oxford Centre for Human Brain Activity, University of Oxford, Oxford, United Kingdom; 2 School of Psychology, University of Birmingham, London, United Kingdom; IIT - Italian Institute of Technology, Italy

## Abstract

By 2015, there will be an estimated two billion smartphone users worldwide. This technology presents exciting opportunities for cognitive science as a medium for rapid, large-scale experimentation and data collection. At present, cost and logistics limit most study populations to small samples, restricting the experimental questions that can be addressed. In this study we investigated whether the mass collection of experimental data using smartphone technology is valid, given the variability of data collection outside of a laboratory setting. We presented four classic experimental paradigms as short games, available as a free app and over the first month 20,800 users submitted data. We found that the large sample size vastly outweighed the noise inherent in collecting data outside a controlled laboratory setting, and show that for all four games canonical results were reproduced. For the first time, we provide experimental validation for the use of smartphones for data collection in cognitive science, which can lead to the collection of richer data sets and a significant cost reduction as well as provide an opportunity for efficient phenotypic screening of large populations.

## Introduction

Innovations such as large-scale genotyping, cohort studies and clinical records linkage allow the analysis of unprecedented amounts of data from exceedingly large numbers of research participants. ‘Big data’, although in principle noisier and less well controlled than small-scale laboratory studies, has the potential to uncover subtle effects such as individual differences, temporal trends and the influence of lifestyle and demographic factors on performance.

In cognitive neuroscience, the conventional paradigm is laboratory-based recruitment of extremely modest sample sizes. Here we show the feasibility and power of a new method of data collection. We developed an app named ‘The Great Brain Experiment’ (www.thegreatbrainexperiment.com) for smartphones that enabled participants to perform four standard experimental paradigms presented in the guise of short games. We hypothesised that the large sample size afforded by this form of mobile data collection would outweigh the problems inherent in collecting data outside a controlled laboratory setting. We also focused on making the experiments quick and enjoyable to complete, in order to maximise the number of completed plays to offset the smaller amount of data that was collected with each play. Here we present results from the app using four established paradigms to demonstrate the feasibility, validity and power inherent in this novel form of large scale cognitive science data collection. A strong consideration in using this type of platform was the need to deploy tasks that were both enjoyable and engaging. These four paradigms were chosen out of many potential tasks because they cover a range of cognitive domains (perception, action inhibition, decision-making and short-term memory), they are easily contextualised within an enjoyable and competitive game framework, and the experimenters had extensive experience in their use under laboratory conditions.

## Materials and Methods

### The app

**Figure 1:**
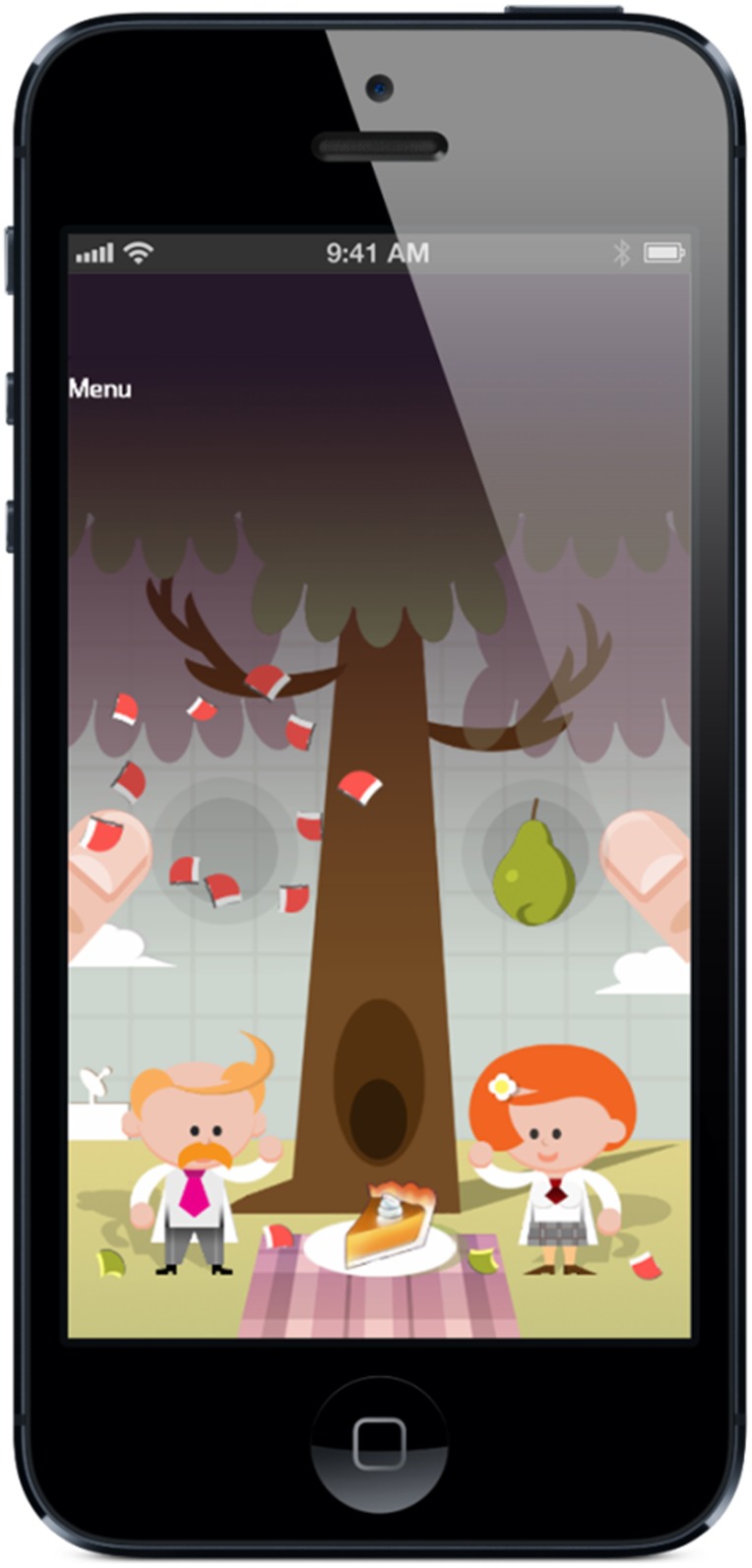
Screenshot of the app, while playing the stop-signal reaction time game. Fruit fell from the tree and participants were asked to tap simultaneously on both sides of the screen as the fruit passed through the circles. If a piece of fruit turned brown during its fall, participants had to inhibit their response on that side.

Initial experimental designs were devised by the study authors, and the games were built for smartphone by an external developer (White Bat Games) (fig. 1). The app was launched for iPhone and Android in the middle of March 2013. Publicity was garnered through blog posts and a number of print articles. The social media sharing function within the app generated word-of-mouth publicity.

Ethical approval for this study was obtained from University College London research ethics committee, application number 4354/001. On downloading the app, participants filled out a short demographic questionnaire and provided informed consent before proceeding to the games. Each time a participant started a game, a counter recording the number of plays was incremented. At completion of a game, if internet connectivity was available, a dataset was submitted to the server containing fields defining the game's content and the responses given. The first time a participant completed any game the server assigned that device a unique ID number (UID). All further data submissions from that device, as well as the demographic information from the questionnaire, were linked to the UID. No personal identification of users was possible at any time.

To maintain interest and enthusiasm in the app, participants could compare their scores against those of other users. They could also read some information about the background and significance of each psychological paradigm.

### Working Memory Task

In the working memory (WM) game, participants were asked to remember the positions of red circles that appeared in different positions on a 4×4 grid. Each trial ended with the presentation of an empty grid and participants were asked to click on the positions in which the red circles had appeared. There were various conditions, but here we focus on two. In the “no distraction” condition the grid containing the red circles appeared for 1 s, followed by a delay period during which the empty grid was displayed for 1 s before the app would accept the participant's response. The “distraction” condition was identical except that two yellow circles (distractors) were presented in the grid during the delay period. The number of red circles (WM load) increased in line with performance (one red circle was added each time a trial of that condition was successfully completed). When a participant failed on two successive trials of a certain condition, the game continued without that condition. The WM load of the last successfully completed trial was used as a measure of performance. Data was removed from 832 participants who failed two successive trials of WM load 2 in any condition, leaving data from 8987 participants. We focussed on participants aged 18–29 years (“young adults”; N = 3247) and 50–69 years (“older adults”; N = 1281). The extent to which participants were affected by the distractors (“distraction cost”) was determined by calculating the percentage difference in performance between the two conditions.

### Attentional blink task

In the attentional blink task, participants were required to identify the second of two target images in a rapid serial visual presentation (RSVP) [1]. The experimental screen showed a pull-down projection screen, an old-fashioned projector in the foreground, and a cartoon scientist character indicating that participants should pay attention to the projection screen. Each trial started with a fixation cross, displayed in the centre of the projection screen for 400 ms. The RSVP contained 14 images. The first target image (T1) was displayed at serial positions 3–7, and the second target image (T2) followed it by 1–5 serial positions (lags 1–5). Images were taken from a stock photo website and were cropped to a square shape and converted to sepia tone. Images fell into one of seven categories - people, chairs, trees, flowers, llamas, fruit and birds. Target images were identified as members of a particular category; the instruction at the beginning of each trial was ‘Watch for the second [category]!’. The rest of the RSVP consisted of random non-target images, with the stipulation that images from the same category could not fall within 3 serial positions of each other. At the end of the RSVP, a fixation cross was presented followed by a screen repeating the instruction and offering a choice of four images from the target category (not including T1). Participants tapped on the image to give their answer.

The ISI between images was 199 ms, 166 ms, 133 ms or 99 ms in the four consecutive levels. These values were determined by the temporal resolution of the smartphone devices, some of which have a maximum refresh rate of 33 ms. Each level consisted of 10 trials; two each at each T2 lag. A score of less than 40% (25% representing chance) in any level terminated the game early. Target categories and images were randomised.

10,503 users completed 14,907 plays in total. Plays which were terminated early were removed from the data, leaving 9,749 users who completed 12,522 plays in total. Proportion correct was calculated for each time lag and each ISI (level). P-values were obtained by calculating the z-test for a proportion, using the formula:
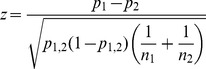
where 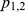 is the total proportion correct across the two test proportions.

### Selective stop-signal task

The stop-signal task measures inhibitory ability. Participants were presented with two pieces of fruit at the top of the screen. After a delay sampled from a uniform distribution between 1000 and 3000 ms, both fruits started to fall towards the bottom of the screen. Participants were instructed to tap both sides of the screen as fruit passed over a shaded area, which corresponded to a window of 500–800 ms after onset of the fall. Upon responses within the time window a green checkmark appeared to indicate a successful trial. Out of 32 trials in the experiment, a random draw of 12 trials (37.5%) were ‘selective stop trials’ on which one of the fruits turned brown, indicating the corresponding side of the screen should not be tapped. Erroneous responses (outside the response window, lack of response, or tapping a bad fruit) were followed by appropriate feedback (‘You touched too soon!’, ‘You touched too late!’, ‘Touch the fruit inside the circles!’, ‘Don't touch the bad fruit!’). On 16 out of 32 trials a glowing circle around one of the fruits indicated to the participant only that fruit could turn brown that trial (which it would do in 6 out of 16 trials, i.e. 37.5%). As such, some trials contained extra information concerning the action that might require stopping, allowing the participant to prepare for a selective stop. This contrasts with the other 16 trials in which no hint was given, and which do not allow for proactive, selective control [2]. Effects related to these differential cues were not examined for this paper. The time at which the fruit turned bad started at 300 ms after onset of falling, and was increased by 50 ms upon a successful stop, and reduced by 50 ms after a failed stop (i.e. commission error). Such a staircasing procedure leads, on average, to 50% success on stop trials [3]. We used separate staircases for trials with and without information on the potential stopping target. Information cues and stop signals were counterbalanced over left and right.

Data collected from the same UID was concatenated, and we discarded data from participants with no correct Go or Stop trials or no failed Stop trials, as estimation of the stop-signal reaction time (SSRT) is either impossible (with no correct Go trials), or unreliable (with 0 or 100% successful stop trials). This left 10,773 out of 12,003 participants, or 90%. We computed the SSRT using the quantile method [3], [4]. All Go reaction times (RTs) were arranged in descending order. The RT corresponding to the participant's probability of successfully stopping over all stop trials was selected (e.g. for a p(stop) of 0.42 we selected the RT 42% down the ordered list). From this value we subtracted the mean time at which the fruit turned bad relative to onset of the start of the fall to obtain the SSRT. As used throughout the literature, this value represents the time it takes for the participant to successfully respond to the stop signal and withhold a response. A fast SSRT, then, allows a participant to inhibit their response even if the fruit turns bad close to the onset of the response. In contrast, higher SSRTs have been associated with impulse control disorders [3], [4].

### Decision-making task

The decision-making task allowed us to measure economic preferences including loss aversion, i.e. greater sensitivity to potential losses than equivalent gains. Participants started with 500 points and made 30 choices between certain outcomes and lotteries with potential gains and losses displayed as numbers of points on the left and right side of a circular spinner. Gambles were chosen by tapping the spinner which spun for 4.4 s before stopping on the outcome. Participants were also asked every 2–3 trials to answer ‘How happy are you right now?’ by marking a point on a line, making 12 ratings including one at the beginning and end of the task. In each play there were 11 gain-only trials (a choice between a points gain and a gamble with a larger potential gain or 0), 11 loss-only trials (a choice between a certain loss or a gamble with a larger potential loss or 0), and 8 mixed trials (a choice between 0 points or a gamble with a gain or loss). Trials were randomly drawn from lists of 60 gain-only trials, 60 loss-only trials, and 30 mixed trials. The gain-only trials list consisted of 4 certain amounts {30,35,45,55} and gamble gain amounts were determined by a multiplier on the certain amount varying from 1.63 to 4 selected to accommodate a wide range of risk sensitivity. The loss-only trials list consisted of 4 certain amounts {−30, −35, −45, −55} and the same multipliers as the gain-only trials list. The mixed trials list consisted of 3 gamble gain amounts {40,55,75} and the corresponding loss amounts determined by a multiplier on the gain amount varying from 0.5 to 5 to accommodate a wide range of loss sensitivity. Participants could gain or lose up to 220 points in a single trial. Our task design and model estimation procedure were similar to a prior study [5]. 9,799 participants (5,839 female) completed the task with 3,463 participating more than once (range, 1–187 plays). We estimated risk aversion and loss aversion using a nonlinear choice model where the utility u(x) of each gain amount x was computed as x^ρ^ and the utility of each loss amount was computed as −λ(−x)^ρ^ where ρ captures risk sensitivity and λ captures loss sensitivity. The experimental design accommodated a range of risk sensitivity from approximately ρ = 0.5–1.4 and a range of loss sensitivity from approximately λ = 0.5–5. The probability that the participant chose the gamble was computed using the softmax function as:
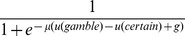
where μ is the sensitivity of choice probability to the utility difference and g captures an overall bias to gamble independent of option values. We estimated parameters for each subject using the method of maximum likelihood.

## Results

### User demographics

**Figure 2:**
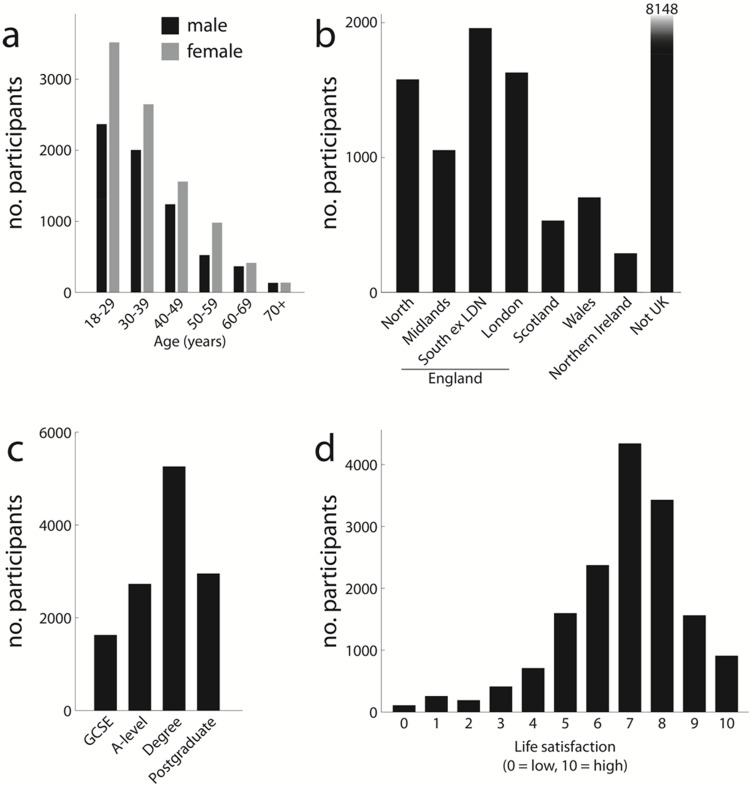
Demographic characteristics of app users. (a) Gender and age breakdown. Young women were the primary app users. (b) Location. Most users originated from outside the UK, where at app was developed, and users from the UK were not concentrated in any single region. (c) The app reached participants with higher education degrees as well as those without. Only participants over 25 years of age were included in this analysis as those younger than 25 may not have completed their education. (d) Life satisfaction rated on a scale from 0–10. This information was recorded for follow-up analyses in relation to the decision-making task and is not further analysed here.

In the first month after release, 44,373 users downloaded the app and 20,800 users (8,355 male) played at least one game to completion and submitted data (approximately 5 minutes). Here we present data from participants over 18 years of age (16,233 users). Upon installing the game, users provided demographic information (age, sex, education, location, and a rating of overall life satisfaction (fig. 2)). 25% used the Android version of the app, the rest used an identical version for iPhone and iPad.

Data from a small fraction of games were lost due to early termination or lack of internet coverage at the time of game completion. For the working memory task, users had started an average of 1.177 games by the time they submitted their first score, according to the app's internal counter; for the stop signal task, the average number of games before submission was 1.225; for the attentional blink it was 1.123 and for the decision-making task this number was 1.107.

### Working memory task

**Figure 3:**
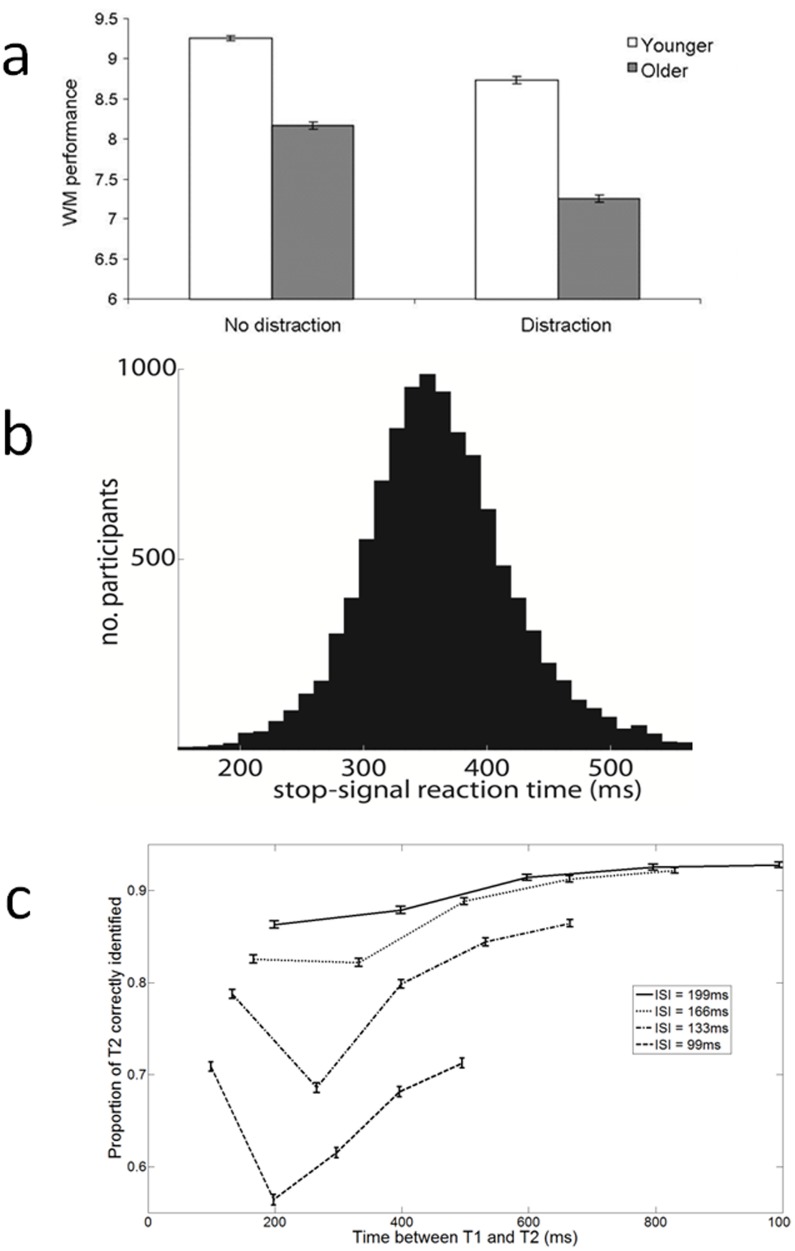
Data from the games in this app. (a) Working memory performance in ‘no distraction’ (remember red circles) and ‘distraction’ (remember red circles and ignore yellow circles) conditions, for younger and older participants. Performance deteriorated with age and distraction, and distraction had a more detrimental effect for older compared to younger adults. (b) Stop-signal reaction time, which measures inhibitory ability, could be estimated from participant's data. (c) Probability of successful identification of T2 in the attentional blink task, for each ISI and lag. T2 recognition was significantly impaired 150–500 ms after T1 presentation, but preserved T2 recognition at lag 1 (‘Lag-1 sparing’) was noted for shorter ISIs.

Performance was significantly lower when distractors were included (F_1,4526_ = 893.97, p<0.001, η_p_^2^ = 0.165). As expected [6], [7], WM performance decreased with age (F_1,4526_ = 1221.33, p<0.001, η_p_^2^ = 0.213) for both conditions (no distraction: t_1848_ = 24.68, p<0.001, Cohen's d = 0.929; distraction: t_2122_ = 29.00, p<0.001, Cohen's d = 1.007, fig. 3). Furthermore, there was a significant interaction between age and condition (F_1,4526_ = 65.80, p<0.001, η_p_^2^ = 0.014), such that older adults were more adversely affected by distractors (mean distraction cost for YA: 4.89%; for OA: 9.23%; t_1889_ = −6.12, p<0.001, Cohen's d = 0.227), supporting the idea of a decline in distractor filtering ability with age [8], [9].

Considering only the younger group, there was a significant difference between the distraction and no distraction conditions (t_3246_ = 21.77, p<0.001, Cohen's d = 0.418). This result replicates a laboratory study [10] in which 21 participants (ages 20–29) performed both distraction and no distraction conditions. Performance was greater for the “no distraction” condition, although the difference did not reach significance (t_20_ = 1.87, p = 0.076, Cohen's d = 0.370).

### Selective stop-signal task

Our data satisfy a prediction of the independent race model [11], the most widely used method for analysis of stop-signal data: stopFail RTs are shorter than Go RTs and thus represent the fast part of the entire Go RT distribution (stopFail RT < Go RT, t_10772_ = 57.8, p<0.001, Cohen's d = 0.56). The effect size was considerably lower than that collected in a similar task under laboratory conditions (Cohen's d = 1.81) [12], possibly reflecting the small number of data points from which the RT measures were derived. We calculated the stop-signal reaction time (SSRT) using the quantile method, which is a robust approach that accounts for inter-individual variability in probability of successful stopping. The SSRT was relatively high compared to the literature (mean (SD): 361.9 (67.7) ms) (fig. 3), indicating participants were relatively slow to inhibit their responses. This potentially reflects the lack of training in our participants, or the uncontrolled environment in which the task was performed. However, the standard deviation of scores was not increased [13], [14]. Note that the SSRT in selective inhibition is known to be longer than in global inhibition, contributing to the relatively high SSRT reported here [15].

### Attentional blink task

The proportion of correct responses for each serial position and each lag is shown in fig. 3. Overall 74% of trials elicited a correct response. For all ISIs, identification of the T2 was significantly impaired at lags 2–4 compared with lag 5 (all p<0.001). ‘Lag 1 sparing’, preserved performance when T2 directly follows the T1 [16], was with ISIs of 133 ms and 99 ms (both p<0.001).

Potter et al. [17] report T2 response independently of T1 detection. They report a lag 5 accuracy of 82% and a 20-percentage-point difference between lag 5 and lag 2, at an ISI of 120 ms. Linear interpolation suggests that from our data we would expect a lag 5 accuracy of 73% and 13-percentage-point difference between lag 5 and lag 2 at this ISI.

### Decision-making task

Participants finished the experiment with mean (SEM) 571.4 (2.2) points, more (p<0.001) than a random strategy player (mean, 514 points). Participants chose the option with the higher expected payoff 60.6 (2.2)% of the time, significantly more than chance (p<0.001). We fit choice data with a model where the parameter λ captures the degree of loss aversion and loss averse participants have λ>1. The mean model fit pseudo-r^2^ = 0.378. The mean loss aversion parameter in our participants was λ = 1.376 (0.013), indicating that the group was loss averse on average. Additional model parameter estimates were µ = 0.241 (0.003), ρ = 0.955 (0.003), and g = 0.822 (0.012). For 3,463 subjects who played more than once, we estimated λ separately for first and second plays and estimates were correlated across the two plays (Spearman's ρ = 0.25, p<0.001) despite the small number of choices in each play, suggesting that the model captures stable difference in economic preferences across our population, allowing participants who are more or less loss averse to be distinguished.

The degree of loss aversion was similar to in a comparable laboratory-based study [5], which reported mean loss aversion λ = 1.40 (0.15) for 30 subjects. Participants in that laboratory experiment each completed 140 choices, more than most of our participants. Examining just our first 60 (of 9,799) participants, who completed an average of 60 trials (2 plays) each, yields a similar estimate of loss aversion: λ = 1.35 (0.15), with an identical variance to the laboratory sample.

## Discussion

These data demonstrate that canonical experimental results can be replicated using smartphone games, despite the relatively uncontrolled environment when compared to laboratory testing. We present data from 16,233 participants, gathered over one month, representing a sixteen-fold increase in the rate of data collection over a previous attempt at smartphone data collection. We speculate this increase might potentially be due to the ‘gamification’ of the experimental paradigms [18] and efforts to package the app in a stylish, engaging format. The app was extensively discussed on Twitter and reviewed favourably on blogs such as the Wall Street Journal Speakeasy blog [19], recruiting further participants. We believe this capturing of attention through social media, which was enabled by making the app both attractive and presenting it as a citizen science project, was responsible for bringing the app to the attention of a large proportion of the eventual users.

Citizen science projects have harnessed the goodwill of internet users to undertake complex data analysis, such as classifying the shapes of galaxies [20], finding optimal protein folding configurations [21], tracking neurons through the retina [22] and deciphering archived manuscripts [23]. Other authors have used Mechanical Turk [24], a service which allows crowdsourcing of short computer-based tasks, to generate human psychological and psychophysical data. They have similarly found that effect sizes are relatively uncompromised [25]. While web-based (e.g. Mechanical Turk) experiments are usually longer and do not require an experimenter to render the tasks engaging, they carry a major disadvantage in so far as their cost scales with the number of participants, whereas the costs of a smartphone app are fixed by the cost of development. It is worth noting that the distinction between our app and Mechanical Turk is the recruitment and motivations of the participants rather than the platform – we could easily create a web-based version of our app, and this could be explored in later developments of the project. However, the use of a smartphone-specific UID theoretically allows for more reliable longitudinal and cross-study data linkage, as data from multiple timepoints and tasks can be associated with a single user with a reasonable level of certainty. In future additions to the app, the wide range of functionality offered by smartphones, including cameras, motion sensors and location-tracking abilities, could potentially be exploited further. Previous work has usefully exploited these characteristics of smartphones in the form of experience sampling [26], and this could usefully be combined with behavioural tasks in future work.

### Accuracy of smartphones

What are the practical limitations of today's smartphones for use in cognitive science experiments? Consistent delivery of visually presented stimuli and collection of timed responses depends on a multitude of factors, key considerations being the performance of the smartphone's screen, processors and careful engineering of the experimental software.

Here, as is typical in computer games design, a central block of software (the ‘game loop’) is executed by the smartphone at high frequency (hardware manufacturers and software developers recommend up to 60 Hz [27], [28]). Each time it is executed, the game loop prepares any changes to the stimuli on screen, and delivers them to the graphics hardware for rendering and display. It is straight forward, therefore, for the experimenter to guarantee the minimum display time for each stimulus: the game loop displays the stimulus, and then it continues looping without making changes to the screen until a predetermined number of seconds has elapsed.

There is, however, an inherent level of inaccuracy in the maximum display time for each stimulus. The precision of this is determined by the performance of the smartphone. For instance, if the game loop operates at 60 Hz, maximum stimulus display times will be accurate to 16.67 ms (1/60 seconds). If the smartphone can only run the game loop at 30 Hz, the maximum stimulus display time will be accurate to 33.33 ms (1/30 seconds). Additional inaccuracies may occur if the experiment requires particularly rapid display of stimuli, and if the smartphone cannot prepare the stimuli quickly enough. To avoid these problems we did not include experiments with ultra-fast stimulus durations (the fastest being the attentional blink task, with stimuli delivered up to 10.1 Hz). Note also that these issues are not specific to smartphones, and also apply to regular computer-based stimulus delivery.

### Effect size comparison

The motivation for carefully controlled laboratory studies is that they increase the effect size of the effect of interest, reducing the number of participants needed to demonstrate the effect. One concern about the use of smartphones is that they will reduce the effect size below the level which can be compensated for by recruitment of additional participants. However, this was not the case for our experiments.

Effect size was not substantially smaller than for a study performed in the laboratory for the working memory task. In this case, although the effect sizes are comparable, the larger number of participants using the app allowed demonstration of the significance of a subtle effect not seen in the laboratory.

Effect size was substantially reduced for the stop-signal task, however this was more than compensated for by the increased sample size. A typical laboratory sample size is 16, which gives 80% power to detect an effect size of d = 1 (a very large effect) at the p = 0.05 two-tailed level. Even if the effect was reduced to just d = 0.2 (an effect of only marginal interest) by translation to smartphones – a much more dramatic decrease in effect size than that seen here – the sample size required to give the same power would be 400, which represents only 4% of the sample sizes achieved by this app.

Comparison of effect sizes in the attentional blink task was challenging because most studies in the literature report the percentage of T2 correctly identified given correct identification of T1, rather than unconditional T2 identification, in order to control for trial-to-trial fluctuations in attention. This partly explains the (apparently) much more dramatic attentional blink effects seen in conventional studies [1]. However, our attentional blink effect was somewhat reduced even when compared with unconditional T2 reports in laboratory studies [2]. The reduction of the attentional blink effect is likely to be caused by the more general reduction in performance, presumably due to increased distraction in the non-lab environment. Asking for a report of T1 might reduce this problem, but would make the game less playable.

In the decision-making task, increased experimental noise might be expected to increase the variance around parameter estimates. However, the population loss aversion parameter could be estimated with similar variance to the laboratory estimate after a smaller total number of plays, suggesting that the uncontrolled environment had little or no effect on behaviour in this game.

These data suggest that apps have real potential to uncover small and subtle psychological effects which could not easily be captured in the laboratory, even under ideal experimental conditions. Future work must explore smartphones' ability to find more subtle cognitive effects before they can be fully validated as data-gathering tools. The loss of power is not as significant as might be expected, and can be easily recouped with the much higher potential participant numbers.

### Demographics of the users

The users recruited were of a broader range of ages, education and geographic location to subjects typically recruited for laboratory experiments from the local University subject pool. 85% of users registered on the subject pool are current university students; of the app users, only 65% of users aged above 24 had completed a degree. 91% of subject pool participants are aged below 32, compared with 35% of app users aged below 30. In addition, only London-based participants can be recruited through the subject pool, whereas the app was downloaded across the UK and internationally.

This approach therefore shares the documented advantages of Web-based research – e.g. the far larger sample size and cost-efficiency, the greater variation amongst participants, etc – and some of the disadvantages, e.g. higher dropout rates and hence a need for shorter and simpler experiments, and the potential lack of one-to-one mapping of users to devices. The statistical power afforded by the former can compensate for the latter in web-based research [29], and we have demonstrated that this is likely to also be the case for smartphone experiments.

### What experiments can be translated to smartphone games?

All four of the experiments we chose had to carefully compromise between obtaining good experimental data and providing an enjoyable user experience. An initial consideration was that the strategy which produced optimal experimental data should be congruent with the category giving a high score. For example, in the stop-signal reaction time experiment the effect size will be maximal when participants are performing as well as they can as differences between performance will not be masked by general increases in reaction time caused by lack of attention or motivation. Attentive, motivated playing is also rewarded by a high points score, meaning the participant is incentivised to produce good experimental data. This might not be the case in, for instance, an experiment looking at the perception of visual illusions, where participants could maximise their points by feigning veridical perception once they understood the nature of the illusion. This is particularly important given the potential non-naiveté of participants [30].

We aimed to ensure that the average time to complete each game was less than 5 minutes, and we observed that the quickest game (the stop-signal reaction time) yielded, and continues to yield, the highest number of plays. Each game was played twice by at least 1,500 participants, indicating that incentives to play multiple times might be an effective way of increasing the size of individual datasets.

All games were extensively piloted for pace and difficulty. We chose to introduce two of the games – attentional blink and working memory – with a very easy level which did not yield much interesting data due to ceiling effects. However, starting with a very easy level reduces the need for complicated explanations of the experiment and encourages participants to persevere with the game. Forced delays were kept to a minimum to maintain attention and interest.

In short, a successful smartphone experiment will be short, fast-paced, easy at the beginning, and performing the experiment in line with the experimenter's objectives will be rewarded with a high points score.

### Limitations of smartphone experiments

The clear potential of smartphone experiments is to gather data from a large number of potentially very diverse participants, and link datasets across time and tasks. However, the use of a smartphone app must be carefully considered. Although direct experimenting time was eliminated in this project, the recruitment and retention of participants required commitment to maintain the high profile of the app through both traditional and social media. Development of the app constituted a fixed investment of time and money, which resulted in good value compared to traditional laboratory experiments because of the number of participants recruited. However, if fewer participants are anticipated to be recruited, or fewer are needed, a web-based or in-house study might prove more time- and cost-effective. The development of an app is substantially more technically specialised than producing a similar experiment in dedicated psychophysics software, meaning the process likely needs to be outsourced, increasing development time (though reducing direct work by the experimenter). However, adding new games to an existing app is a much simpler process, reducing costs in all these domains.

Smartphone experiments will never be able to offer guaranteed one-to-one mapping of users to devices. Confidentiality issues and incomplete internet coverage mean some data will always be lost, while the potential for distracting factors is greatly multiplied compared to in-house experiments. The limitation on the length of games necessarily limits the precision of any individual subject estimates, while variation in the technical specifications of smartphone models might mean smartphones are not a suitable medium for certain psychophysical experiments where physical stimulus properties are important.

Nonetheless, smartphones are a unique avenue through which members of the general public can be engaged with and participate in scientific research. Research funding organisations are increasingly recognising the importance of ‘public engagement’ – informing and exciting people outside of the scientific community about scientific research. Apps appeal to an increasing public interest in science and an increasing desire to actively participate in science, and we are hopeful that they can also help build trust and mutual understanding between researchers and the public.

### Conclusion

We are currently extending the capabilities of the app, adding further experiments in the auditory and motor domains, as well as allowing researchers to invite participants for laboratory-based research based on their performance in the app. We suggest apps could thus be used as a screening tool for studies that aim to characterize extremes of the population. These participants might then be further assessed using neuroimaging techniques.

There are currently over one billion smartphones in use worldwide, and this number is predicted to rise to two billion by 2015 [31]. Smartphone users represent a participant pool far larger and more diverse than could ever be studied in the laboratory. In time, data from simple apps such as this one might be combined with medical, genetic or lifestyle information to provide a novel tool for disease risk prediction and health monitoring, in addition to helping uncover the links between psychological characteristics, demographics, and wellbeing.
