# Correction: Crowdsourcing for Cognitive Science – The Utility of Smartphones

**DOI:** 10.1371/journal.pone.0111159

**Published:** 2014-10-14

**Authors:** The PLOS ONE Staff

There are errors in the author affiliations. The affiliations should appear as shown here:

Harriet R. Brown^1,3^, Peter Zeidman^3^, Peter Smittenaar^3^, Rick A. Adams^3^, Fiona McNab^2,3^, Robb B. Rutledge^3^, Raymond J. Dolan^3^

1 Oxford Centre for Human Brain Activity, University of Oxford, Oxford, United Kingdom, 2 School of Psychology, University of Birmingham, Birmingham, United Kingdom, 3 Wellcome Trust Centre for Neuroimaging, University College London, London, United Kingdom
